# Thieme MedOne ComSci

**DOI:** 10.5195/jmla.2018.470

**Published:** 2018-07-01

**Authors:** Marilia Y. Antunez

**Affiliations:** Science and Technology Library, The University of Akron, Akron, OH

## GENERAL DESCRIPTION

Part of Thieme’s MedOne online package, MedOne Communication Sciences (MedOne ComSci) is a small database of publications in communication science and disorders, designed to be a teaching and learning resource for upper-level undergraduate students, graduate students, faculty, and practicing professionals in audiology, speech-language pathology, and hearing science. Consisting of four sources (e-books, cases, e-journals, and media), the database is well organized, and the content is relatively current. This product represents the first attempt to combine full-text searchable books, cases, journals, and media from a single publisher in a unifying platform covering the field of communicative disorders.

**Figure 1:**
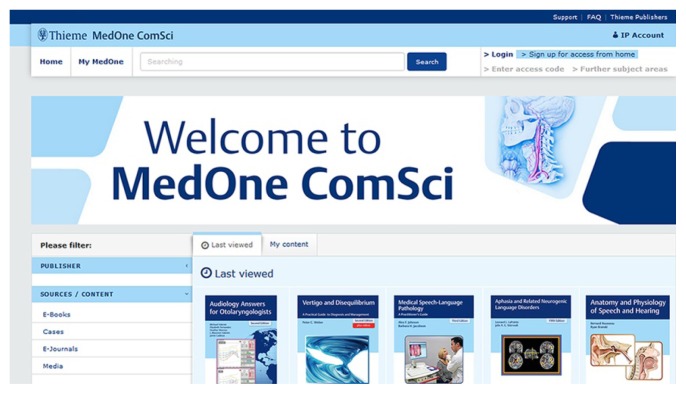
Thieme MedOne ComSci home page

Formerly called Thieme eCommunication Science [1], the product was redesigned in November 2017. The redesigned product features the standard MedOne home page, with a colorful header informing users of new or forthcoming cases, e-books, and a featured “case of the week” (Figure 1). Users can search all of MedOne’s ComSci online content in one single search box and limit results to e-books, media, e-journals, or PubMed abstracts. Another new feature lets users track what they have recently reviewed on the “Last viewed” tab or in “My content,” which shows all content that is available to the user. Since the new design, two new e-books have been added, but otherwise most of the content remained the same.

## CONTENT

### E-books

A major feature of MedOne ComSci is its e-book collection. For instance, most of the cases and media files come from e-books in the database. As of February 2018, forty Thieme e-books were available. As new and revised editions are released (typically an average of three to four books annually [2]), they are automatically added to the collection. The collection includes some well-known textbooks in the field, such as *Medical Speech-Language Pathology: A Practitioner’s Guide* and *Essentials of Audiology.*

The Thieme e-books cover different aspects of the speech-language pathology and audiology fields (e.g., hearing aids, practice management), and publication dates range from 2000 to 2018 editions. Some are Doody Core Title List (DCT) titles [3], and no open access textbooks are included. One dictionary and a few other reference works (e.g., handbooks) are included. Libraries with existing online Thieme subscribers (e.g., MedOne Education) benefit by integrating content since the product does not include all Thieme e-books in the field of speech-language pathology, audiology, and peripheral fields (e.g., otolaryngology). When searching e-books, users can browse by title, author/editor, or publication date. The e-books are digital rights management (DRM)–free and have unlimited simultaneous users.

### Journals

Two peer-reviewed Thieme journals, *Seminars in Hearing* and *Seminars in Speech and Language,* are available in full-text with coverage beginning in 2013. According to the vendor, journal coverage lags about two months behind the journal website [4]. However, when the product was tested for this review in December 2017 and January 2018, the most recent articles were from March 2017. Users can browse journals by title, year, and issue. Another option is to select quick links to Continuing Education Self-Study Program, Preface, or Review Article from these journals. Articles include additional readings for further research. When the product was tested for this review, it did not include digital object identifiers (DOIs) for books or articles, although vendor staff later reported that DOIs for articles have been added on the MedOne platform [2]. Thieme is planning to add DOIs for e-books [4].

### Cases

There were 190 audiology cases and 86 cases in speech-language pathology at the time of this review, and more than half were pediatric cases. A few cases were listed in both the speech-language pathology and audiology categories (e.g., Down syndrome, cytomegalovirus). Key features of the cases (adult and pediatric) are the Questions for the Reader and Discussion of Questions sections. These sections provide pertinent questions including follow-up tips and answers to the questions discussed in the chapters. Other case sections include a description of the problem and recommended treatment, outcome, key points, and suggested readings. As with all content in MedOne ComSci, users can easily access specific sections in the cases.

### Media

Media content includes audio, static images, and video. As of this writing, the product included 46 audio files, 5,696 images, and 55 videos. Images included figures, (e.g. audiograms, photographs), tables, and some color images. Users can enlarge images using full-screen mode and compare multiple images by displaying them side by side. A link is available for each image to allow users to easily request assistance from Thieme. All media content includes a legend with persistent links to the original source.

There are some limitations related to media content in MedOne ComSci. Several small images of equations were difficult to view online and in printed form due to their low resolution. Authenticated users can download, print, and share images offline for educational use only, but video and audio files cannot be downloaded. MedOne platforms cannot be integrated with learning management systems, (i.e., content cannot be embedded or downloaded) [2].

## USER INTERFACE

The interface is clean and easy to navigate. Users can search and browse by either selecting a category (E-Books, Cases, E-Journals, and Media), selecting a subject area (Audiology, Otolaryngology, and Speech Language Pathology), or searching in a single search box that searches all MedOne ComSci content plus PubMed. The search function supports Boolean operators, phrase searching, and truncation. Despite a few inconsistencies in retrieved results, relevant results were included in the top three search results for each category when sample searches were performed for this review. The first three search results are displayed separately in each category or collection, along with the first five PubMed search results at the abstract level. Search terms are highlighted on the search results page. Search results can be sorted by date or relevance and filtered by category or subject area.

## ACCESSIBILITY AND USABILITY

Links to an FAQ and support are available in all web page headers. When reading, users can magnify text size by clicking on the plus or minus buttons. A “Reader Mode” is available, which offers a simpler screen with no page header. Images and videos can be viewed in full screen mode. Headings or sections of documents are hyperlinked for easier access to specific content. Users can highlight selected text, create notes, and save the information automatically in their “My MedOne” accounts. Navigation would be easier if links to next and previous pages were available at the top as well as the bottom of the page. Perhaps most concerning, closed captioning is not available, a serious limitation for institutions that are required to comply with accessibility standards.

The database allows users to export bibliographical information in RIS format for use with citation management software. A persistent link (called a short link by Thieme) is available for online web pages and documents. An option to email or print citations in American Psychological Association (APA) citation style would be useful, as that is the style that most audiology and speech-language pathology degree programs require. When citing or printing full text, users should use the “Open PDF view” option rather than the “Open Print (PDF) view” option, as the former includes page numbers, while the latter does not.

## AUTHENTICATION AND TECHNICAL REQUIREMENTS

MedOne ComSci is supported by all major browsers (Firefox, Chrome, Safari, and Internet Explorer 9 or higher), and it is optimized for both phone and tablet devices [2]. Institutional access to MedOne ComSci is authenticated by Internet protocol (IP) address. Users at licensed institutions must create a Thieme personal account to access the database remotely. Subscribed institutions have access to MARC records for e-books only. MedOne ComSci was expected to begin offering COUNTER usage statistics and reports for e-books in April 2018 [4].

## RECOMMENDATIONS

MedOne ComSci’s single-publisher model fills a niche that is not found in the current publishing market for the multidisciplinary fields of speech-language pathology and audiology. Other platforms that provide content from different vendors (e.g., ProQuest Ebook Central, R2 Digital Library, STAT!Ref) usually do not include full-text journal content and might not include some unique e-books that are only available from Thieme. Other publishers covering the areas of communication disorders (e.g., Jones & Barlett Learning [5], Plural Publishing [6]) also provide instruction resources similar to MedOne ComSci; however, these publishers either provide content in print only or, if they offer online content, it consists of electronic journals only.

While Thieme materials are well respected because of their authoritative content and are used by many faculty for instruction, MedOne ComSci is not comprehensive in coverage and scope, because it does not include content from other publishers in the field. Compared to related online resources, the breadth of coverage is small in MedOne ComSci, with only two journals and fewer than one hundred e-books. The product also lacks some features that are common in other online content platforms and even other MedOne products (e.g., interactive quizzes). Librarians should consider whether this product duplicates holdings in other platforms or in their print collections.

MedOne ComSci is worth considering by libraries serving students, faculty, and researchers in communication science and disorders. Currently, no other products on the market contain Thieme content in these disciplines. Despite being a small database, MedOne ComSci provides a mix of online resources in the areas of speech-language pathology and audiology in one convenient platform for online learning.
